# Comparative Analysis of Complete Chloroplast Genome Sequences in *Edgeworthia* (Thymelaeaceae) and New Insights Into Phylogenetic Relationships

**DOI:** 10.3389/fgene.2021.643552

**Published:** 2021-03-10

**Authors:** Shaojuan Qian, Yonghong Zhang, Shiou Yih Lee

**Affiliations:** ^1^School of Life Sciences, Yunnan Normal University, Kunming, China; ^2^State Key Laboratory of Biocontrol and Guangdong Provincial Key Laboratory of Plant Resources, School of Life Science, Sun Yat-sen University, Guangzhou, China

**Keywords:** chloroplast genome, comparative analysis, *Edgeworthia*, internal transcribed spacers, phylogenetic relationship, Thymelaeaceae

## Abstract

The complete chloroplast genomes of three species of *Edgeworthia* namely, *Edgeworthia albiflora*, *Edgeworthia chrysantha*, and *Edgeworthia gardneri* (Thymelaeaceae), are reported and characterized. The chloroplast genomes displayed a typical quadripartite structure with conserved genome arrangement and specific divergence. The genomes ranged in length from 172,708 to 173,621 bp and displayed similar GC content of 36.5–36.7%. A total of 138–139 genes were predicted, including 92–93 protein-coding, 38 tRNAs and eight rRNAs genes. Variation in the number of short simple repeats and inverted region boundaries of the three cp genomes were observed. A mutational hotspot was detected along the nucleotide sequence from the *ndh*F to the *trn*L-UAG genes. The chloroplast genome-based and internal transcribed spacer (ITS)-based phylogenetic analyses using maximum-likelihood (ML) and Bayesian inference (BI) revealed that *E. albiflora* diverged before *E. chrysantha* and *E. gardneri* and placed the *Edgeworthia* clade at the base of the Eurasian Daphne group with strong bootstrap support. With an effective taxonomic treatment of the species of *Edgeworthia*, further molecular analyses of their intra- and interspecific genetic variation are inclined to support the treatment of *E. albiflora* and *E. gardneri* as two natural groups. The genetic information obtained from this study will provide valuable genomic resources for the identification of additional species and for deducing the phylogenetic evolution of *Edgeworthia*.

## Introduction

The family Thymelaeaceae is composed of about 900 species in 45 genera. The most recently proposed taxonomic classification based on palynological findings divided the family into two major subfamilies, Octolepidoideae and Thymelaeoideae. The latter consists of three tribes: Aquilarieae, Daphneae, and Synandrodaphneae (Herber, 2003). The Daphneae accounts for the most genera, which were further clustered into four different groups, Daphne, Gnidia, Linostoma, and Phaleria, with the Daphne group containing the most genera.

As a member of the Daphne group, *Edgeworthia* is reported to contain five species; *E. albiflora* Nakai, *E. chrysantha* Lindl., *Edgeworthia eriosolenoides* K. M. Feng & S. C. Huang, *E. gardneri* (Wall.) Meisn., and *E. longipes* Lace. They occur naturally in China, India, and nearby regions (The Plant List, 2013; Wang and Gilbert, 2017). Studies of the phytochemical and pharmacological properties of *Edgeworthia* have received much attention among researchers (Nan et al., 2018), as the inflorescences and stems of *E. chrysantha* and *E. gardneri* are regarded as effective folk medicines for muscle relaxation and to treat rheumatism (Xiao, 2002; Che et al., 2010). Rich in low-lignin fibers and their ease of propagation, plants of *Edgeworthia* are not only cultivated as ornamentals in urban areas (Clennett et al., 2002; Wang et al., 2017), but are also the preferred raw material for high quality paper products, such as banknotes, and artificial cotton production (Lan et al., 2013). For molecular information, genetic studies on *Edgeworthia* were confined to the molecular placement of *E. chrysantha* within the Thymelaeaceae using short gene sequences (Van der Bank et al., 2002; Beaumont et al., 2009; Motsi et al., 2010; Foster et al., 2016), while genetic information for other species of *Edgeworthia* is limited.

The chloroplast (cp) genome is responsible for photosynthesis (Leister, 2003; Wicke et al., 2011). In general, the cp is maternally inherited and consists of a quadripartite circular double-stranded DNA molecule that comprises two copies of inverted repeat (IRs) regions, a large single-copy (LSC) region, and a small single copy (SSC) region (Palmer, 1985). The length of a typical cp genome ranges between 120,000 and 160,000 bp but variations can occur. Due to its relatively small size, simple structure, conserved gene content, and order, cp genome sequences have been widely used in phylogenetic studies and provide valuable data for resolving complex evolutionary relationships (Jansen et al., 2007; Moore et al., 2010).

At present, there are only 25 complete cp genomes for taxa in the family Thymelaeaceae available publicly in the GenBank database (as of 1st December 2020), with *Aquilaria sinensis* as the first taxon reported (Wang et al., 2016). Due to the lack of molecular information on the genus *Edgeworthia*, we used next-generation sequencing technology to obtain the complete cp genomes of three species of *Edgeworthia*, including *E. albiflora*, *E. chrysantha*, and *E. gardneri*. We constructed and characterized the cp genome structure of these species and performed phylogenetic analyses at the genome-scale level. In addition, to expand the genomic resources from these valuable species, we also sequenced the nuclear ribosomal DNA internal transcribed spacer (ITS) region to reveal the phylogenetic relationships of these species of *Edgeworthia* to other closely related taxa in the Daphne group.

## Materials and Methods

### Plant Materials and DNA Extraction

Fresh leaf samples from three species of *Edgeworthia* namely, *E. albiflora*, *E. chrysantha*, and *E. gardneri*, were collected from plants in their natural habitat and were stored in Ziplock bags filled with silica gel beads prior to transportation to the laboratory. Voucher specimens of the three species were deposited in the Herbarium of Yunnan Normal University (YNUB) (Table 1). Total genomic DNA was extracted from the silica-dried leaves using the modified cetyltrimethylammonium bromide (CTAB) method (Doyle and Doyle, 1987) and was further purified using Wizard^®^ DNA Clean-Up System (Promega, United States).

**TABLE 1:** Basic characteristics of chloroplast genomes in three species of *Edgeworthia*.

	***E. albiflora***	***E. chrysantha***	***E. gardneri***
Sample location	Miyi, Sichuan, China	Kunming Botanical Garden, Yunnan, China	Motuo, Tibet, China
Collector and collection number	Zhang Y.; ZYH11	Zhang Y.; RXK26	Zhang Y.; RXK53
Chloroplast genome GenBank accession number	MW246180	MN511715	MW246181
ITS GenBank accession number	MW255615	MW255616	MW255617
LSC (bp)	86,862	85,824	86,388
SSC (bp)	2,681	2,816	3,017
IR (bp)	42,039	42,034	41,952
Total (bp)	173,621	172,708	173,309
Protein-coding genes	93	93	92
tRNA genes	38	38	38
rRNA genes	8	8	8
Total	139	139	138
GC content (%)	36.5	36.7	36.5

### Genome Sequencing, Assembly and Annotation

Next-generation sequencing was conducted on the Illumina HiSeq 2500 platform and a 350-bp paired-end library was prepared. The raw reads were filtered to obtain high-quality clean reads using NGS QC Toolkit v2.3.3 with default parameters (Patel and Jain, 2012). The cp genome was assembled using NOVOPlasty (Dierckxsens et al., 2017) with the *rbc*L gene of *Daphne kiusiana* Miq. extracted from the complete cp genome sequence (GenBank accession KY991380) as the seed sequence. The complete cp genome was annotated using Geneious v10.1.3 (Kearse et al., 2012) by referring to the cp genome sequence of *D. kiusiana*. Annotations on the protein-coding (CDS) sequences present in the genome were manually checked using the open reading frame (ORF) and the tRNA genes were verified using the online tRNAscan-SE web server with default settings (Lowe and Chan, 2016). The complete cp genome was visualized using OGDRAW v1.3.1 (Greiner et al., 2019); all cp genome sequences were deposited in the NCBI GenBank database under the accession numbers MW246180 (*E. albiflora*), MN511715 (*E. chrysantha*), MW246181 (*E. gardneri*) for future reference.

### Repeats Analyses

Simple sequence repeats (SSRs) were identified using the MISA-web (Beier et al., 2017). The minimum number of repeats was set at 10, 5, 4, 3, 3, and 3 for mono-, di-, tri-, tetra-, penta-, and hexanucleotides, respectively. SSRs were manually checked for redundancy. Identification of the four different types (forward, palindromic, reverse, and complement) of large repeats were conducted with REPuter (Kurtz et al., 2001). The size and identity of the large repeats were limited to not less than 30 bp and 90%, respectively; while the Hamming distance was set at 3.0.

### Genome Comparison

The junctions and borders of the inverted repeat (IR) regions were visualized using IRscope (Amiryousefi et al., 2018) and further edited using Adobe Photoshop CS6 (Adobe, United States). For comparative analysis, the three sequences of the *Edgeworthia* cp genomes were compared using mVISTA (Mayor et al., 2000) using Shuffle-LAGAN mode. The cp genome sequence of *E. albiflora* was selected as the reference genome. The output image was manually edited using Adobe Illustrator 2020 (Adobe, United States). All three genome sequences of the *Edgeworthia* cp were aligned using MAFFT v7.409 (Katoh and Standley, 2013). Highly divergent regions between the species were identified using DnaSP v5.10 (Librado and Rozas, 2009). The nucleotide divergence values of the cp genome sequence alignment were analyzed using the sliding window method, with a window length of 1000 bp and a 500-bp step size.

### Codon Use Preference Analysis

All the CDS gene sequences were manually extracted from the chloroplast genome. The codon usage frequency in each of the three species of *Edgeworthia* was analyzed for all the PCGs using MEGA5 (Kumar et al., 2008). The relative synonymous codon usage (RSCU) was conducted to determine if the plastid genes were under selection.

### Polymerase Chain Reaction and Sanger Sequencing

Polymerase chain reaction (PCR) amplification was conducted on a final reaction volume, with a 20 μL volume reaction consisting of 10 μL of 2× Taq PCR StarMix with loading dye (Genstar Biosolutions, China), 1 μL of each primer, 6 μL of distilled water, and 2 μL 5 ng genomic DNA as a template. The ITS universal primer set: ITS1, 5′-TCC GTA GGT GAA CCT GCG G-3′ (forward) and ITS4, 5′-TCC TCC GCT TAT TGA TAT GC-3′ (reverse), was used to obtain the ITS region (White et al., 1990). PCR amplification was programmed with thermal settings of an initial denaturation at 94°C for 5 min; denaturation at 94°C for 60 s, annealing at 55°C for 60 s, extension at 72°C for 60 s; and a final extension at 72°C for 7 min. Upon verification via electrophoresis on a 1.0% agarose gel and documented under the UV machine, the PCR products were sent for direct Sanger sequencing at both ends using an ABI 3730 DNA Analyzer (Applied Biosystems, United States). Results acquired from the Sanger sequencing were aligned and manually edited to obtain the clean sequences of the three species of *Edgeworthia*. The ITS sequences for *E. albiflora* (MW255615), *E. chrysantha* (MW255616), and *E. gardneri* (MW255617) were deposited in the NCBI GenBank database for future reference.

### Phylogenetic Analyses

Complete cp genome sequences of 15 taxa from the family Thymelaeaceae were included for phylogenetic analyses using maximum likelihood (ML) methods and Bayesian inference (BI). Multiple sequence alignment was carried out using MAFFT v7.409 (Katoh and Standley, 2013). Based on the Akaike information criterion calculated from the Modeltest 3.7 (Posada and Crandall, 1998), the generalized-time-reversible (GTR) model with gamma (+G) and invariant sites included (+I) (=GTR + G + I) was the best-fitting substitution model for both the ML and BI analyses. The ML tree was constructed using RAxML 8.2.11, under 1,000 bootstrap replicates (Stamatakis, 2014). BI analysis was conducted using MrBayes 3.2.5 (Drummond and Rambaut, 2007), in which the Markov Chain Monte Carlo analysis was performed under 1,000,000 generations and four Markov chains. Samplings were conducted at every 1,000 generations. The first 25% of the trees was discarded as burn-in; the remaining trees were estimated using the 50% majority-rule consensus tree and Bayesian posterior probabilities. Two closely related species, *Hibiscus hamabo* (Malvaceae; KR259988) and *Eugenia uniflora* (Myrtaceae; KR867678) were included as outgroups.

A total of 23 ITS sequences from the members of Thymelaeaceae, representing 21 taxa from eight genera in the Daphne group of tribe Daphneae, two taxa from tribe Aquilarieae and one taxon from the subfamily Octolepidoideae, were retrieved from the NCBI GenBank database. The latter three taxa were then selected as outgroups. Along with the ITS sequences of the three *Edgeworthia* species, the sequences were MUSCLE-aligned using MEGA 5 (Kumar et al., 2008) and trimmed using trimAL v1.2 (Capella-Gutiérrez et al., 2009) with the gappyout method in order to reduce the systematic errors produced by poor alignment. Phylogenetic analyses were carried out using both the ML and BI method. For ML analysis, the optimal DNA substitution model for the ML analysis calculated using the “Find Best DNA/Protein Model (ML)” function embedded in MEGA 5 (Kumar et al., 2008) was the Kimura two-parameter (K2P) with discrete Gamma model (+G) and invariant included (+I) (=K2P + G + I). Calculation was conducted with 1,000 bootstrap replicates on each branch node and all gaps and missing data were included in the analysis. For BI analysis, calculation was performed using MrBayes v3.2.5 (Drummond and Rambaut, 2007) following the same parameters and settings as mentioned above.

## Results

### Chloroplast Genome Features

A total of 23,660,708 raw reads were obtained and the raw reads were directly fed into the assembly pipeline to obtained the maximum amount of useful data. Prior to genome assembly, a total of 374,342 aligned reads were acquired, and 222,318 assembled reads of an average coverage depth of 217 times per site were incorporated in the genome assembly. Three contigs representing three species of *Edgeworthia* were obtained at the end of the assembly process.

**FIGURE 1:**
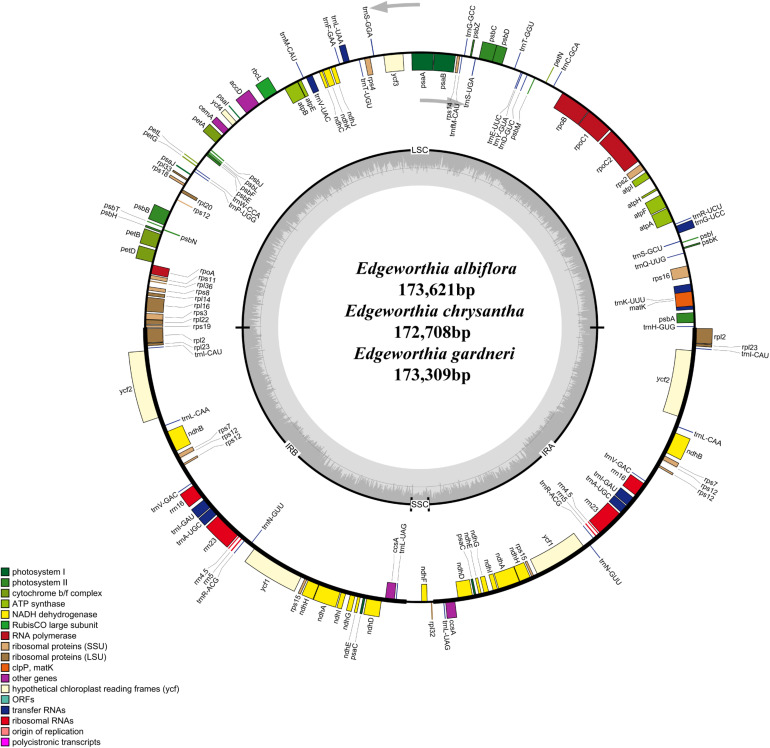
Gene map for chloroplast genomes of three species of *Edgeworthia*. Annotated genes are colored according to functional categories. Genes placed outside the outer circle were transcribed clockwise; genes placed inside the circle were transcribed counterclockwise. Dark gray in inner circle represents GC content whereas light gray corresponds to AT content.

The cp genomes of the species of *Edgeworthia* were typical quadripartite structures that ranged in size from 172,708 bp (*E. chrysantha*) to 173,621 bp (*E. albiflora*) (Figure 1). All genomes contained a pair of IRs (41,952–42,039 bp), separated by a large single-copy (LSC) region (85,824–86,862 bp) and a small single-copy (SSC) region (2,681–3,017 bp) (Table 1). A total of 139 genes were predicted for the cp genomes of *E. albiflora* and *E. chrysantha*, including 93 CDS genes, 38 transfer RNA (tRNA) genes, and eight ribosomal RNA (rRNA) genes. However, only 138 genes were recorded in the cp genome of *E. gardneri*, in which only 92 CDS genes, 38 tRNA genes, and eight rRNA genes were predicted. In the LSC region, *E. albiflora* and *E. chrysantha* each had 61 CDS genes, but only 60 CDS genes were reported in *E. gardneri.* The CDS gene, *cem*A, was presumably a pseudogene in the cp genome of *E. gardneri*; while only two CDS genes were recorded in all three species of *Edgeworthia*. All three cp genomes of *Edgeworthia* had 27 genes replicated in both IR regions, including 15 CDS genes (*ccs*A, *ndh*A, *ndh*B, *ndh*D, *ndh*E, *ndh*G, *ndh*H, *ndh*I, *rpl*2, *rpl*23, *rps*12, *rps*15, *psa*C, *ycf*1, and *ycf*2), eight tRNA genes (*trn*A-UGC, *trn*I-CAU, *trn*I-GAU, *trn*L-CAA, *trn*L-UAG, *trn*N-GUU, *trn*R-ACG, and *trn*V-GAC), and four rRNA genes (*rrn*4.5, *rrn*5, *rrn*16, and *rrn*23) (Table 2). Of the 19 intron-containing genes, 17 of them (*atp*F, *ndh*A, *ndh*B, *pet*B, *pet*D, *rpl*2, *rpl*16, *rpo*C1, *rps*16, *trn*A-UGC, *trn*G-UCC, *trn*I-GAU, *trn*K-UUU, *trn*L-UAA, and *trn*V-UAC) contained one intron, whereas two of them (*rps*12 and *ycf*3) contained two introns (Table 3). The GC content of *E. chrysantha* and *E. albiflora* was 36.5%, while the GC content of *E. chrysantha* was 36.7%.

**TABLE 2:** Gene contents in three *Edgeworthia* species chloroplast genome.

**Classification**	**Genes**
**Self-replication**	
Large ribosomal subunits	rpl2*(×2), rpl14, rpl16*, rpl20, rpl22, rpl23(×2), rpl32, rpl33, rpl36
Small ribosomal subunits	rps2, rps3, rps4, rps7, rps8, rps11, rps12**^a^(×2), rps14, rps15(×2), rps16*, rps18, rps19
DNA dependent RNA polymerase	rpoA, rpoB, rpoC1*, rpoC2
Ribosomal RNAs	*rrn4.5(×2), rrn5(×2), rrn16(×2), rrn23(×2)*
Transfer RNAs	trnA-UGC*(×2), trnC-GCA, trnD-GUC, trnE-UUC, trnF-GAA, trnfM-CAU, trnG-GCC, trnG-UCC*, trnH-GUG, trnI-CAU(×2), trnI-GAU*(×2), trnK-UUU*, trnL-CAA(×2), trnL-UAA*, trnL-UAG(×2), trnM-CAU, trnN-GUU(×2), trnP-UGG, trnQ-UUG, trnR-ACG(×2), trnR-UCU, trnS-GCU, trnS-GGA, trnS-UGA, trnT-GGU, trnT-UGU, trnV-GAC(×2), trnV-UAC*, trnW-CCA, trnY-GUA
**Photosynthesis-related genes**	
Photosystem I	*psaA, psaB, psaC(×2), psaI, psaJ*
Photosystem II	*psb*A, *psb*B, *psb*C, *psb*D, *psb*E, *psb*F, *psb*H, *psb*I, *psb*J, *psb*K, *psb*L, *psb*M, *psb*N, *psb*T, *psb*Z
NAD(P)H dehydrogenase complex	*ndh*A*(*×*2), *ndh*B*(*×*2), *ndh*C, *ndh*D(*×*2), *ndh*E(*×*2), *ndh*F, *ndh*G(*×*2), *ndh*H(*×*2), *ndh*I(*×*2), *ndh*J, *ndhK*
F-type ATP synthase	*atp*A, *atp*B, *atp*E, *atp*F*, *atp*H, *atp*I
Cytochrome b6/f complex	*pet*A, *pet*B*, *pet*D*, *pet*G, *pet*L, *pet*N
Rubisco	*rbc*L
**Other genes**	
Envelope membrane protein	*cem*A
Maturase	*mat*K
Cytochrome c biogenesis protein	*ccs*A(*×*2)
Subunit of acetyl-CoA-carboxylase	*acc*D
**Unknown**	
Conserved open reading frames	*ycf*1(*×*2), *ycf*2(*×*2), *ycf*3**, *ycf*4

*Gene containing one intron, **gene containing two introns, ^*a*^*trans-*splinting gene, (×2) shows gene shave two copies.

**TABLE 3:** Locations and sizes of the intron-containing genes recorded in the chloroplast genome of the three *Edgeworthia* species.

**Species**	**Gene**	**Location**	**Exon I (bp)**	**Intron I (bp)**	**Exon II (bp)**	**Intron II (bp)**	**Exon III (bp)**
*Edgeworthia albiflora*	*atp*F	LSC	144	864	411	na	na
	*ndhA*	IR	558	1165	540	na	na
	*ndh*B	IR	777	686	756	na	na
	*pet*B	LSC	6	770	698	na	na
	*pet*D	LSC	8	786	475	na	na
	*rpl*2	IR	393	724	391	na	na
	*rpl*16	LSC	9	1080	399	na	na
	*trn*A-UGC	IR	38	812	35	na	na
	*trn*G-UCC	LSC	23	720	49	na	na
	*trn*I-GAU	IR	37	955	35	na	na
	*trn*K-UUU	LSC	37	2559	35	na	na
	*trn*L-UAA	LSC	34	546	50	na	na
	*trn*V-UAC	LSC	38	615	35	na	na
	*ycf*3	LSC	126	732	228	761	153
*Edgeworthia chrysantha*	*atp*F	LSC	144	867	411	na	na
	*ndh*A	IR	558	1163	540	na	na
	*ndh*B	IR	777	690	756	na	na
	*pet*B	LSC	6	759	698	na	na
	*pet*D	LSC	8	790	475	na	na
	*rpl*2	IR	391	683	434	na	na
	*rpl*16	LSC	9	1066	399	na	na
	*rpo*C1	LSC	432	767	1617	na	na
	*rps*16	LSC	40	934	206	na	na
	*trn*A-UGC	IR	38	811	35	na	na
	*trn*G-UCC	LSC	23	718	49	na	na
	*trn*I-GAU	IR	37	955	35	na	na
	*trn*K-UUU	LSC	37	2555	35	na	na
	*trn*L-UAA	LSC	34	551	50	na	na
	*trn*V-UAC	LSC	38	621	35	na	na
	*ycf*3	LSC	126	732	228	761	153
*Edgeworthia gardneri*	*atp*F	LSC	144	856	411	na	na
	*ndh*A	IR	558	1162	540	na	na
	*ndh*B	IR	777	690	756	na	na
	*pet*B	LSC	6	766	698	na	na
	*pet*D	LSC	8	781	475	na	na
	*rpl*2	IR	393	681	434	na	na
	*rpl*16	LSC	9	1076	399	na	na
	*rpo*C1	LSC	432	757	1617	na	na
	*rps*16	LSC	40	946	200	na	na
	*trn*A-UGC	IR	38	812	35	na	na
	*trn*G-UCC	LSC	23	725	49	na	na
	*trn*I-GAU	IR	37	955	35	na	na
	*trn*K-UUU	LSC	37	2531	35	na	na
	*trn*L-UAA	LSC	34	547	50	na	na
	*trn*V-UAC	LSC	38	620	35	na	na
	*ycf*3	LSC	126	727	228	762	153

*na, not available.*

### Sequence Repeats

**FIGURE 2:**
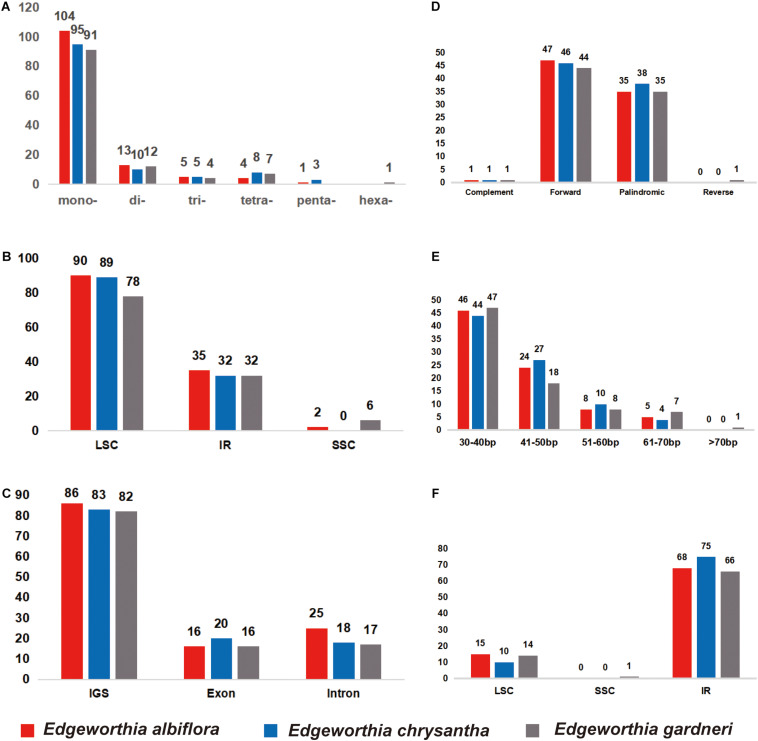
Distribution of small sequence repeats (SSRs) in the chloroplast genomes of three species of *Edgeworthia*. **(A)** Number of different SSR types detected in chloroplast genomes of three species of *Edgeworthia*; **(B)** Frequencies of identified SSRs in large single-copy (LSC), small single-copy (SSC), and inverted repeats (IRs) regions; **(C)** Frequencies of identified SSRs in the gene coding and intergenic region. **(D)** Frequencies of four different large repeat types (complement, forward, palindromic, and reverse) in the chloroplast genomes of three species of *Edgeworthia*; **(E)** Frequencies of large repeats based on different sequence length (bp) groups; **(F)** Frequencies of large repeats in the LSC, SSC, and IRs regions.

Simple sequence repeat analysis detected 127, 121, and 115 SSRs in *E. albiflora*, *E. chrysantha*, and *E. gardneri*, respectively (Figure 2A). Most of the SSRs were in the LSC regions when compared to the SSC and IR regions (Figure 2B). The SSRs were more abundant in the intergenic spacer region when compared to both the intronic and exon regions; more than 80 SSRs were detected in the intergenic spacer regions of the three species of *Edgeworthia*, and the number of SSRs in the intronic and exon regions were only recorded between 16 and 25 (Figure 2C). Cp genomes of all three *Edgeworthia* species contained mono-, di-, tri-, and tetranucleotide SSRs; while *E. albiflora* and *E. chrysantha* had one and three pentanucleotide SSRs, respectively, none were recorded in *E. gardneri*. Yet, *E. gardneri* was recorded with one hexanucleotide SSR, which was not present in the other two species. Considering sequence complementary, eight classified repeat types were found present in all three species of *Edgeworthia* (data not shown). The repeat type C/G were only detected in *E. albiflora* and *E. chrysantha*; while the repeat type AATT/AATT were only detected in *E. chrysantha* and *E. gardneri*. The repeat types AAG/GTT and ACAT/ATGT were exclusive to *E. chrysantha*, and *E. gardneri* was the only species recorded with the repeat types AATTC/AATTG and ACCCC/GGGGT.

For large repeats, forward repeats were recorded most abundant in the cp genome of the three *Edgeworthia* species, ranging from 44 to 47, followed by the palindromic repeats that ranged from 35 to 38 (Figure 2D). However, there was no records for reverse repeats in *E. albiflora* and *E. chrysantha*, but one in *E. gardneri*. The large repeats were recorded mostly in sequence length of 30–40 bp (Figure 2E). *Edgeworthia gardneri* was recorded with one large repeat with sequence length over 70 bp, but only large repeats with length from 60 to 70 bp were recorded longest for *E. albiflora* and *E. chrysantha*. The large repeats were recorded mostly distributed in the IR region, followed by the LSC region; at least one large repeat was recorded in the SSC region in *E. gardneri* (Figure 2F).

### Contraction and Expansion of the IR Regions

**FIGURE 3:**
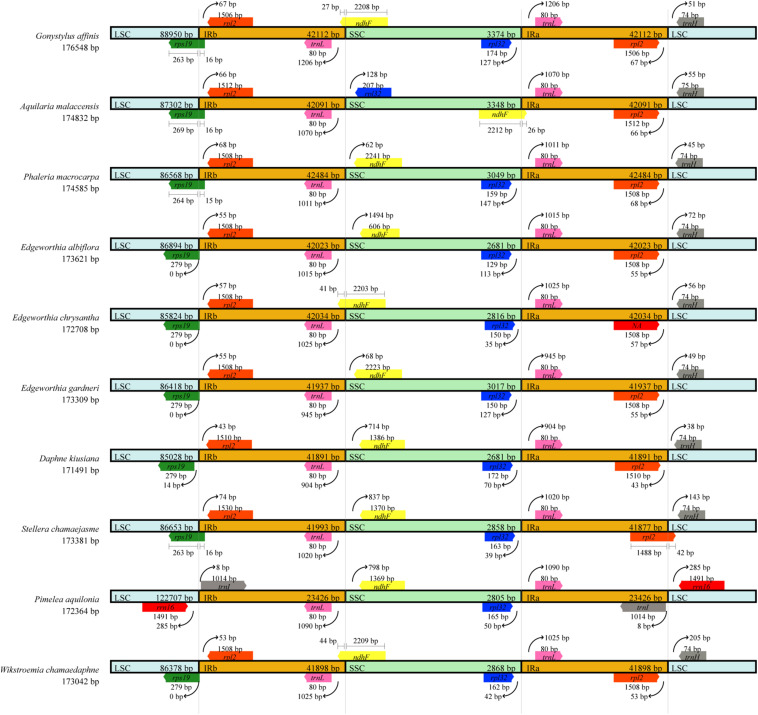
Comparison of borders between large single-copy (LSC), small single-copy (SSC), and inverted repeat (IR) regions across ten chloroplast genomes of Thymelaeaceae.

Chloroplast genome structure and the junction positions between IR regions were well-conserved among the three species of *Edgeworthia*, but structural variation was present in the IRs/SC borders (Figure 3). The *ndh*F gene extended to the IRB region in the cp genome of *E. chrysantha*, but not for *E. albiflora* and *E. gardneri.* The *ndh*F gene in the latter two species was located in the SSC region. The *rps*19 gene that was located in the LSC region in *E. chrysantha* extended into the IRB region in *E. albiflora* and *E. gardneri*. When compared to other seven closely related genera in the family Thymelaeaceae, the placement of genes adjacent to the IR junctions were identical to those in the cp genome of *Gonystylus affinis*, *D. kiusiana*, *Phaleria macrocarpa*, *Stellera chamaejasme*, and *Wikstroemia chamaedaphne*.

### Comparative Genomic Analysis

**FIGURE 4:**
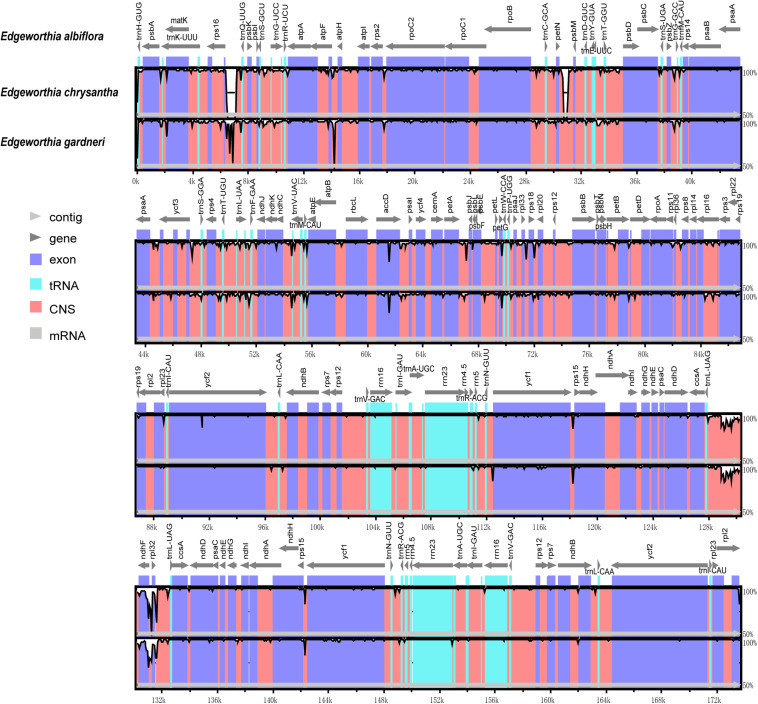
Complete chloroplast genome comparison of three species of *Edgeworthia* using mVISTA alignment program. Genome regions are color-coded and described in the figure legend.

**FIGURE 5:**
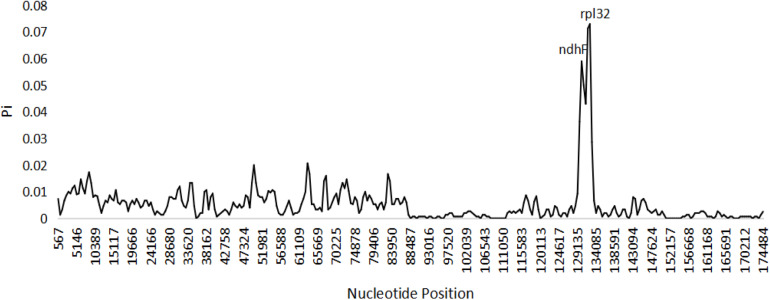
Sliding window analysis of the complete chloroplast genome sequences among three species of *Edgeworthia* (Window length: 1000 bp; step size: 500 bp).

Based on the genome sequence alignment of the three species of *Edgeworthia*, distinct sequence variation was detected in three gene regions; *pet*N-*psb*M, *trn*L-UAG-*rpl*32, and *rps*16-*tr*nQ-UUG (Figure 4). With a nucleotide diversity (Pi) cut-off point set at Pi ≥ 0.04, the sliding window analysis detected three highly variable regions in the genome sequence alignment of the three species of *Edgeworthia* (Figure 5). The highly variable regions were all located within the protein-coding genes, *ndh*F and mainly manifested in the SSC region, between *ndh*F and *rpl*32 of the cp genome.

### Codon Use Preference Analysis

A total of 29,529–30,093 codons of the CDS genes were recorded in the three cp genomes of *Edgeworthia*. The RSCU value for each species exhibited similar codon preference in the 64 codons in the CDS genes (Supplementary Table 1). As a result, 30 of them exhibited greater preference (RSCU > 1); 32 of them were least preferred (RSCU < 1); two of them displayed no preferences (RSCU = 1). The isoleucine (Ile)-encoded codon AUU exhibited the greatest occurrence (*n* = 1,269); while the Ile-encoded codon UGA exhibited the least occurrence (*n* = 20). Among the preferred codons, 27 of them were A/U-ended. Among the three stop codons, UAA was recorded to be more abundant than UAA and UGA, thus displaying higher preferences. There were no rare codons (RSCU < 0.1) found in the CDS genes of the three cp genomes of *Edgeworthia*.

### Phylogenetic Analysis

**FIGURE 6:**
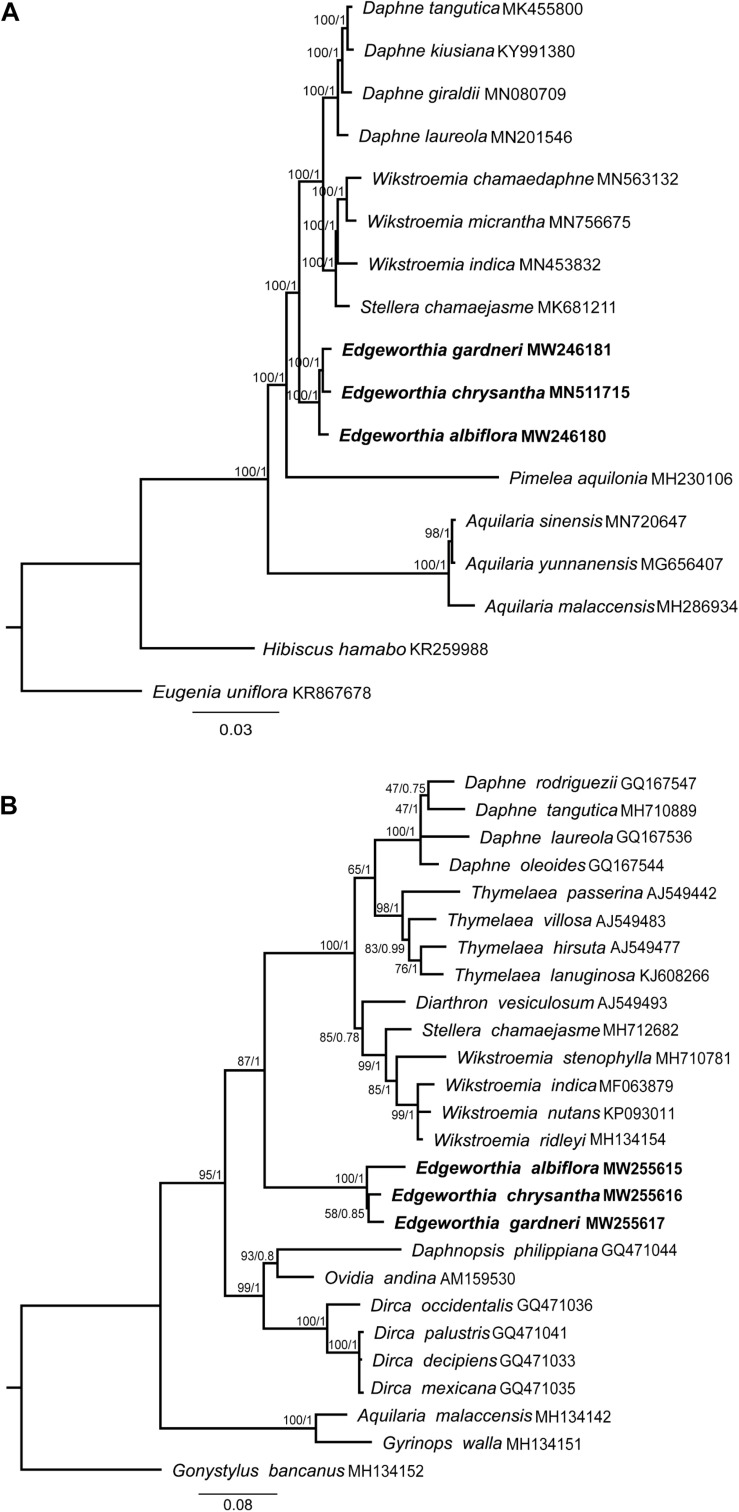
Maximum-likelihood (ML) and MrBayes (BI) tree analyses were based on 1,000 bootstrap replicates. Bootstrap support values and Bayesian posterior probabilities are indicated at each branch nodes. Three species of Edgeworthia used in this study are highlighted in bold. **(A)** Combined phylogenetic tree of *Edgeworthia* and allied genera based on the chloroplast genome sequences of 15 taxa from Thymelaeaceae. Two related species, *Hibiscus hamabo* (Malvaceae; KR259988) and *Eugenia uniflora* (Myrtaceae; KR867678) were included as outgroups. **(B)** Phylogenetic analyses of Thymelaeaceae based on nuclear ribosomal DNA internal transcribed spacer (ITS) gene sequences. A total of 23 ITS sequences from the members of Thymelaeaceae, representing 21 taxa from eight genera in the Daphne group of tribe Daphneae, two taxa from tribe *Aquilarieae* and one taxon from subfamily Octolepidoideae.

Phylogenetic analyses using the complete cp genome sequence for both ML and BI methods revealed similar topological structure between the two phylogenetic trees (Figure 6A). Strong bootstrap support and high posterior probabilities were recorded at all branch nodes. All taxa included in this study displayed monophyletic relationships. In the Daphneae, *Edgeworthia* diverged before *Daphne*, *Stellera*, and *Wikstroemia*. The three species of *Edgeworthia* formed a monophyletic clade, with *E. albiflora* diverging before *E. chrysantha* and *E. gardneri*.

For the ITS-based ML and BI analyses, both phylogenetic trees exhibited identical tree structure and species placement (Figure 6B). The Daphne group displayed a paraphyletic relationship, in which the genera *Daphnopsis*, *Dirca*, and *Ovidia* formed a cluster; while *Edgeworthia* was placed at the base of the latter cluster, with strong bootstrap support and Bayesian posterior probability (ML ≥ 75, BI ≥ 0.95) that also consisted of *Daphne*, *Diarthron*, *Stellera*, *Thymelaea*, and *Wikstroemia*. In the *Edgeworthia* clade, the three *Edgeworthia* species formed a monophyletic clade, with *E. albiflora* diverging before *E. chrysantha* and *E. gardneri* under strong bootstrap support and Bayesian posterior probability.

## Discussion

Similar to other members of the Thymelaeaceae, the cp genome of *Edgeworthia* was rather well-conserved across the three species analyzed in this study. By comparing published cp genome reports on members of the Thymelaeaceae, it was determined that the complete cp genome sequence of *Edgeworthia* is shorter than those of *G. affinis* of subfamily Octolepidoideae (176,548 bp) and nine species of *Aquilaria* (*Aquilaria beccariana*, *Aquilaria crassna*, *Aquilaria hirta*, *Aquilaria malaccensis*, *Aquilaria microcarpa*, *Aquilaria rostrata*, *A*. *sinensis*, *Aquilaria subintegra*, and *Aquilaria yunnanensis*; 174,693–174,907 bp) from Aquilarieae of subfamily Thymelaeoideae (Hishamuddin et al., 2020), but was longer than the four species of *Daphne* (*Daphne giraldii*, *D*. *kiusiana*, *Daphne laureola*, and *Daphne tangutica*; 169,944–171,643 bp) (Cho et al., 2018; Könyves et al., 2019; Yan et al., 2019a,b), two species of *Wikstroemia* (*W*. *chamaedaphne* and *Wikstroemia indica*; 151,731–173,042 bp) from the Daphne group (Qian and Zhang, 2020; Qian et al., 2020), and *Pimelea aquilonia* (172,364 bp) from the Gnidia group (Foster et al., 2018), all belonging to the Daphneae of subfamily Thymelaeoideae. *Stellera chamaejasme*, from the monospecific *Stellera*, was the only species from the Daphne group with a total cp genome size within the cp genome size range of *Edgeworthia*, which was 173,381 bp (Yun et al., 2019). Eventually, rather small size of the SSC region was observed in the cp genome of most species from Thymelaeaceae. In general, the expansion, shrinkage and loss of the IR regions are some of the known reasons to variations in cp genome sizes of seed plants (Jansen and Ruhlman, 2012). Since information on complete cp genomes in members of the Thymelaeaceae is limited, we could not infer any significance between the cp genome size and the systematic position in the Thymelaeaceae.

In this study, the three species of *Edgeworthia* shared almost the same number of genes in their complete cp genomes; *E. gardneri* was recorded as having one gene fewer when compared to the other two species – the protein-coding gene, *cem*A, was not annotated. The *cem*A gene of *E. gardneri* was thought to be a pseudogene as the loci of the premature stop codons differ from the others and could be causing the gene to be non-functional; while the location of the stop codons in *cem*A could be useful for distinguishing *E. gardneri* from *E. albiflora* and *E. chrysantha*. The c*em*A gene encodes the chloroplast membrane protein, which may play an important role in plastid maintenance and intracellular communication (Sasaki et al., 1993; Sonoda et al., 1997). The *cem*A homolog is also recorded to be essential for carbon dioxide (CO_2_) transfer in cyanobacteria (Katoh et al., 1996). The stomatal density of *E. gardneri* is lower than that in *E. chrysantha* (Zhang et al., 2015) and the deletion of *cem*A gene may be related to its adaptation in its habitat, which has an average altitude of above 1,000 m a.s.l.

Based on literature reports, members of the Daphne group can be divided into two natural groups due to the effects on geographical separation and by minor morphological differences (Herber, 2003). The seven American genera (*Daphnopsis*, *Dirca*, *Funifera*, *Goodallia*, *Lagetta*, *Ovidia*, and *Schoenobiblus*) have petals and/or long filaments, whereas the seven Eurasian genera (*Daphne*, *Diarthron*, *Edgeworthia*, *Rhamnoneuron*, *Stellera*, *Thymelaea*, and *Wikstroemia*) lack petals, but are reported to have sessile or subsessile anthers (Herber, 2003). Eventually, the most comprehensive phylogenetic study conducted on Thymelaeaceae using the combined *rbc*L, *trn*L-*trn*F datasets and ITS sequences revealed that members of the Daphne group were actually divisible into two different clades in which *Daphne*, *Diarthron*, *Edgeworthia*, *Stellera*, and *Wikstroemia* formed one clade; while *Ovidia* and *Dirca* were in another clade with *Peddiea* (Phaleria group) and *Stephanodaphne* (Linostoma group) (Beaumont et al., 2009). Similarly, a non-monophyletic relationship was observed in the Daphne group in this study based on the ITS sequences. In our study, the three American genera, *Dirca*, *Ovidia*, and *Daphnopsis* formed a clade independent from the Eurasian genera (Figure 6B). Both the phylogenetic analyses based on cp genome sequences and the nuclear ribosomal DNA ITS sequences, or a combination of both the cp and nuclear gene sequences, placed *Edgeworthia* at the base of the Daphne group among the Eurasian genera.

Taxonomic controversy is also present in *Edgeworthia*. Based on the Flora of China, there are at least five species, whereas four species (*E. albiflora*, *E. chrysantha*, *E. eriosolenoides*, and *E. gardneri*) are in the Asia region (Wang and Gilbert, 2017). The recent revision by The Plant List committee recognizes only four species; *E. eriosolenoides*, *E. gardneri*, *E. longipes*, and *Edgeworthia tomentosa* (The Plant List, 2013). The former synonymized *E. chrysantha* with *E. tomentosa*, as the name *E. tomentosa* was considered invalid in a revision conducted on the collections of Thunberg on *Magnolia tomentosa* (Hamaya, 1955), and also *E. albiflora* with *E. gardneri* (Wang and Gilbert, 2017). *Edgeworthia albiflora*, a species treated as a distinct since it was first discovered in 1924 (Nakai, 1924; Duncan and Mellinger, 1972), was later regarded as a synonym of *E. gardneri* (Clennett et al., 2002). The synonymy was not accepted in the Flora of China, where *E. albiflora* was treated as distinct (Wang and Gilbert, 2017). It is noteworthy that we also failed to recover information to synonymize *E. albiflora* under *E. gardneri*. Based on our field observations, *E. albiflora* and *E. gardneri* can be differentiated through their morphological features and do not pose a challenge in species recognition. The interior of the calyx of *E. gardneri* is yellow, the ovary uniformly hairy, the stigma rounded; the interior of the calyx of *E. albiflora* is white, the base of the ovary is glabrous and the apex hairy and the stigma is clavate (Wang and Gilbert, 2017). Meanwhile, the leaf epidermis is entirely different in *E. albiflora* and *E. gardneri*, with paracytic stomatal types and cyclocytic stomatal types, respectively (Zhang et al., 2015). From a molecular perspective, it is generally accepted that species are delimited when the interspecific variation is greater than intraspecific variation (Lim et al., 2012). Thus, to further strengthen the case for recognizing *E. albiflora*, we compared the genetic information and found that the alignment between *E. albiflora*, *E. chrysantha*, and *E. gardneri* is consisted of greater interspecific variation (cp genome: pairwise distance = 0.0045–0.0061, containing 1038 singletons; ITS: pairwise distance = 0.0226–0.0365, containing 29 singletons) than its intraspecific variation that was based on the alignment between our collection of *E. chrysantha* and another published genome of *E. chrysantha* (cp genome: MT135125; ITS: AJ744932) (cp genome: pairwise distance = 0.0004, containing 70 singletons; ITS: pairwise distance = 0.0030, containing two singletons) (data not shown). Furthermore, the molecular placement with strong bootstrap support based on the ML and BI trees using both the complete cp genome sequences and ITS sequence, analyzed separately, revealed that *E. albiflora* and *E. gardneri* should be treated separately (Figure 6B). Unless there is a stronger case to synonymize the two species via morphological features, judging from the molecular evidence and personal field observation, they should be regarded as two natural groups. On the other hand, two species, *E*. *eriosolenoides* and *E. longipes* were not included in this study; no specimens had been collected since they were first described.

## Conclusion

The entire cp genomes of *E. albiflora*, *E. chrysantha*, and *E. gardneri* were sequenced and analyzed in this study. We obtained such comprehensive molecular information as SSRs, IR contraction and expansion, codon usage and phylogenomic placement through explicit bioinformatic analyses of the cp genome. Furthermore, the addition of the ITS sequences for the understudied species of *Edgeworthia* provided insight for the first time on the phylogenetic relationships of the three species of *Edgeworthia* at the nuclear gene level. The data obtained from this study will likely provide a powerful genetic resource for future studies on population genetics, biological functions, molecular phylogeny, as well as evolution of *Edgeworthia*.

## Data Availability Statement

The datasets presented in this study can be found in online repositories. The names of the repository/repositories and accession number(s) can be found below: https://www.ncbi. nlm.nih.gov/genbank/, MW246180; https://www.ncbi.nlm.nih.gov/genbank/, MN511715; https://www.ncbi.nlm.nih.gov/genbank/, MW246181; https://www.ncbi.nlm.nih.gov/genbank/, MW255615; https://www.ncbi.nlm.nih.gov/genbank/, MW255616; and https://www.ncbi.nlm.nih.gov/genbank/, MW255617.

## Author Contributions

SQ performed the experiments, analyzed the data, and wrote the manuscript. YZ and SL conceived the research and revised the manuscript. All authors read and approved the final manuscript.

## Conflict of Interest

The authors declare that the research was conducted in the absence of any commercial or financial relationships that could be construed as a potential conflict of interest.
