# Supplementary material for: Comparative Analysis of Complete Chloroplast Genome Sequences in Edgeworthia (Thymelaeaceae) and New Insights Into Phylogenetic Relationships
Source: Front Genet. 2021 Mar 10;12:643552. doi: 10.3389/fgene.2021.643552 (PMC8006312; doi:10.3389/fgene.2021.643552)
Supplement: Supplementary file 1 [file Table_1.DOCX]

**Table S1.** Relative synonymous codon usage of protein-coding genes in chloroplast genomes of three species of *Edgeworthia.*

| Codon (Amino Acid) | tRNA | *E. albiflora* | |  | *E. chrysantha* | |  | *E. gardneri* | |
| --- | --- | --- | --- | --- | --- | --- | --- | --- | --- |
|  |  | Count | RSCU |  | Count | RSCU |  | Count | RSCU |
| UUU (Phe) | *trn*F-GAA | 1151 | 1.31 |  | 1196 | 1.32 |  | 1187 | 1.33 |
| UUC (Phe) |  | 601 | 0.69 |  | 614 | 0.68 |  | 601 | 0.67 |
| UUA (Leu) | *trn*L-UAA | 1007 | 1.92 |  | 1042 | 1.93 |  | 1037 | 1.95 |
| UUG (Leu) | *trn*L-CAA | 627 | 1.19 |  | 640 | 1.19 |  | 634 | 1.19 |
| CUU (Leu) | *trn*L-UAG | 675 | 1.29 |  | 694 | 1.29 |  | 683 | 1.29 |
| CUC (Leu) |  | 206 | 0.39 |  | 210 | 0.39 |  | 204 | 0.38 |
| CUA (Leu) |  | 428 | 0.82 |  | 436 | 0.81 |  | 421 | 0.79 |
| CUG (Leu) |  | 207 | 0.39 |  | 210 | 0.39 |  | 210 | 0.40 |
| AUU (Ile) | *trn*I-GAU | 1235 | 1.46 |  | 1269 | 1.47 |  | 1258 | 1.47 |
| AUC (Ile) |  | 492 | 0.58 |  | 492 | 0.57 |  | 481 | 0.56 |
| AUA (Ile) | *trn*I-CAU | 805 | 0.95 |  | 826 | 0.96 |  | 828 | 0.97 |
| AUG (Met) | *trn*fM-CAU | 686 | 1.00 |  | 699 | 1.00 |  | 695 | 1.00 |
| GUU (Val) | *trn*V-GAC | 584 | 1.53 |  | 587 | 1.51 |  | 577 | 1.50 |
| GUC (Val) |  | 186 | 0.49 |  | 183 | 0.47 |  | 179 | 0.47 |
| GUA (Val) | *trn*V-UAC | 573 | 1.50 |  | 586 | 1.51 |  | 582 | 1.51 |
| GUG (Val) |  | 188 | 0.49 |  | 198 | 0.51 |  | 199 | 0.52 |
| UCU (Ser) | *trn*S-GGA | 650 | 1.72 |  | 658 | 1.71 |  | 648 | 1.71 |
| UCC (Ser) |  | 352 | 0.93 |  | 360 | 0.94 |  | 356 | 0.94 |
| UCA (Ser) | *trn*S-UGA | 446 | 1.18 |  | 453 | 1.18 |  | 452 | 1.19 |
| UCG (Ser) |  | 215 | 0.57 |  | 225 | 0.59 |  | 216 | 0.57 |
| CCU (Pro) | *trn*P-UGG | 453 | 1.50 |  | 464 | 1.52 |  | 462 | 1.51 |
| CCC (Pro) |  | 235 | 0.78 |  | 235 | 0.77 |  | 235 | 0.77 |
| CCA (Pro) |  | 353 | 1.17 |  | 357 | 1.17 |  | 355 | 1.16 |
| CCG (Pro) |  | 166 | 0.55 |  | 169 | 0.55 |  | 168 | 0.55 |
| ACU (Thr) | *trn*T-GGU | 581 | 1.63 |  | 601 | 1.65 |  | 594 | 1.65 |
| ACC (Thr) |  | 263 | 0.74 |  | 263 | 0.72 |  | 262 | 0.73 |
| ACA (Thr) | *trn*T-UGU | 446 | 1.25 |  | 450 | 1.24 |  | 450 | 1.25 |
| ACG (Thr) |  | 138 | 0.39 |  | 139 | 0.38 |  | 135 | 0.37 |
| GCU (Ala) | *trn*A-UGC | 659 | 1.73 |  | 677 | 1.74 |  | 676 | 1.74 |
| GCC (Ala) |  | 268 | 0.70 |  | 273 | 0.70 |  | 272 | 0.70 |
| GCA (Ala) |  | 428 | 1.12 |  | 442 | 1.13 |  | 434 | 1.12 |
| GCG (Ala) |  | 169 | 0.44 |  | 168 | 0.43 |  | 173 | 0.45 |
| UAU (Tyr) | *trn*Y-GUA | 926 | 1.62 |  | 953 | 1.63 |  | 952 | 1.63 |
| UAC (Tyr) |  | 214 | 0.38 |  | 216 | 0.37 |  | 215 | 0.37 |
| UAA (End) |  | 45 | 1.45 |  | 45 | 1.45 |  | 44 | 1.43 |
| UAG (End) |  | 27 | 0.87 |  | 27 | 0.87 |  | 28 | 0.91 |
| CAU (His) | *trn*H-GUG | 516 | 1.51 |  | 529 | 1.51 |  | 518 | 1.51 |
| CAC (His) |  | 167 | 0.49 |  | 170 | 0.49 |  | 166 | 0.49 |
| CAA (Gln) | *trn*Q-UUG | 807 | 1.54 |  | 819 | 1.54 |  | 809 | 1.54 |
| CAG (Gln) |  | 239 | 0.46 |  | 242 | 0.46 |  | 239 | 0.46 |
| AAU (Asn) | *trn*N-GUU | 1105 | 1.56 |  | 1135 | 1.57 |  | 1124 | 1.57 |
| AAC (Asn) |  | 314 | 0.44 |  | 310 | 0.43 |  | 311 | 0.43 |
| AAA (Lys) | *trn*K-UUU | 1235 | 1.47 |  | 1257 | 1.47 |  | 1249 | 1.47 |
| AAG (Lys) |  | 446 | 0.53 |  | 454 | 0.53 |  | 447 | 0.53 |
| GAU (Asp) | *trn*D-GUC | 936 | 1.55 |  | 941 | 1.55 |  | 934 | 1.56 |
| GAC (Asp) |  | 268 | 0.45 |  | 271 | 0.45 |  | 266 | 0.44 |
| GAA (Glu) | *trn*E-UUC | 1172 | 1.47 |  | 1185 | 1.47 |  | 1176 | 1.47 |
| GAG (Glu) |  | 428 | 0.54 |  | 432 | 0.53 |  | 428 | 0.53 |
| UGU (Cys) | *trn*C-GCA | 246 | 1.42 |  | 254 | 1.43 |  | 252 | 1.42 |
| UGC (Cys) |  | 101 | 0.58 |  | 101 | 0.57 |  | 102 | 0.58 |
| UGA (End) |  | 21 | 0.68 |  | 21 | 0.68 |  | 20 | 0.65 |
| UGG (Trp) | *trn*W-CCA | 519 | 1.00 |  | 530 | 1.00 |  | 519 | 1.00 |
| CGU (Arg) | *trn*R-ACG | 380 | 1.27 |  | 383 | 1.26 |  | 381 | 1.27 |
| CGC (Arg) | *trn*R-UCU | 118 | 0.39 |  | 121 | 0.40 |  | 120 | 0.40 |
| CGA (Arg) |  | 407 | 1.36 |  | 407 | 1.34 |  | 407 | 1.35 |
| CGG (Arg) |  | 125 | 0.42 |  | 129 | 0.43 |  | 127 | 0.42 |
| AGU (Ser) | *trn*S-GCU | 447 | 1.19 |  | 455 | 1.18 |  | 449 | 1.18 |
| AGC (Ser) |  | 153 | 0.41 |  | 156 | 0.41 |  | 154 | 0.41 |
| AGA (Arg) |  | 560 | 1.87 |  | 570 | 1.88 |  | 562 | 1.87 |
| AGG (Arg) |  | 204 | 0.68 |  | 207 | 0.68 |  | 206 | 0.69 |
| GGU (Gly) | *trn*G-GCC | 625 | 1.30 |  | 632 | 1.29 |  | 633 | 1.29 |
| GGC (Gly) |  | 189 | 0.39 |  | 195 | 0.40 |  | 194 | 0.40 |
| GGA (Gly) | *trn*G-UCC | 771 | 1.60 |  | 779 | 1.59 |  | 779 | 1.59 |
| GGG (Gly) |  | 345 | 0.72 |  | 351 | 0.72 |  | 353 | 0.72 |
